# Rapalink-1 reveals an mTOR- and p53-regulated transcriptional program in Neuro-2a cells

**DOI:** 10.1242/bio.062902

**Published:** 2026-10-22

**Authors:** Joel Karikari Nyarkoh, Matthew Wolan, Eric Barrett, Aidan M. Sokolov, Rachel A. Lyman, Trudy F. C. Mackay, David M. Feliciano

**Affiliations:** ^1^ Department of Biological Sciences, Clemson University, Clemson, SC 29634, USA; ^2^ Department of Biochemistry and Genetics, Clemson University, Clemson, SC 29634, USA; ^3^ Institute for Human Genetics, Clemson University, Greenwood, SC 29646, USA

**Keywords:** Rapalink-1, mTORC1, mTOR, Cancer

## Abstract

The mammalian target of rapamycin (mTOR) is the catalytic subunit of two exclusive complexes referred to as mTORC1 and mTORC2. The mTORCs phosphorylate different substrates and control different cellular processes. Inhibitors of mTORC1 include rapamycin analogs (rapalogs) that are useful in many clinical settings. However, they have failed in cancer trials potentially due to resistance mechanisms and incomplete inhibition of mTORC1 phosphorylation of select substrates. The rapamycin sensitivity of mTORC1 substrates is likely encoded by the substrate's primary amino acid sequence determinants and the ability of substrates to fit into the catalytic region of the kinase super-complex. Thus, some mTORC1 substrates are unaffected by rapalogs. Second-generation ATP-binding pocket inhibitors are mTOR catalytic inhibitors that bind in an ATP-competitive manner. Torin1 is an exemplary second-generation mTOR inhibitor with superior inhibition of mTORC1 substrates, including 4EBP. However, these inhibitors fail to demonstrate selectivity between mTORC1 and mTORC2. Third-generation bisteric mTORC1 inhibitors are exemplified by Rapalink-1, which links a rapamycin moiety to the ATP-competitive inhibitor MLN-0128. Here, we report that Rapalink-1 inhibits three mTORC1 substrates, including 4EBP and Ulk1, which are rapamycin insensitive. To provide additional mechanistic insight into the Rapalink-1 mechanism of action, RNA sequencing of Neuro-2a cells treated with Rapalink-1 was performed. Rapalink-1 caused transcriptomic changes indicative of reductions in the mTOR pathway and brain development. Many transcripts contained a p53 binding site, and consistent with this finding, we also observed reduced levels of p53. These results confirm and expand upon the Rapalink-1 mechanism of action.

## Teaser

**Summary:** mTOR inhibition and transcriptomic alterations provide mechanistic insight into the effect of Rapalink-1 on nervous system cancer cell lines

## INTRODUCTION

The discovery of a bacterial macrolide, rapamycin, produced by *Streptomyces hygroscopicus*, facilitated the delineation of a nutrient-sensitive pathway implicated in health and disease (Vézina et al., 1975). Both rapamycin and the structurally similar FK-506 compound are immunosuppressants that work through distinct mechanisms and bind to FKBP12 (Bierer et al., 1990; Dumont et al., 1990). The rapamycin-dependent association of FKBP12 precipitates a protein called RAFT1 (rapamycin and FKBP12 target 1) (Sabatini et al., 1994). This protein, contemporaneously isolated by others, was also called FRAP (mammalian FKBP–rapamycin-associated protein), but the name mammalian target of rapamycin (mTOR) is most used (Brown et al., 1994). mTOR orthologs include yeast *TOR1* (*DRR1*) and *TOR2* (*DRR2*) and are recognized to have highly conserved roles in regulating growth pathways in relation to nutrients (Heitman et al., 1991; Helliwell et al., 1994). Inactivating mutations of the yeast orthologs *FKB12*, *TOR1*, and *TOR2* result in rapamycin insensitivity. In yeast, rapamycin treatment phenocopies *TOR1* deletion and inhibits mRNA translation and cell growth (although not cell size).

mTOR is localized to at least two complexes in mammals, termed mTORC1 and mTORC2 (Kim et al., 2002; Dos et al., 2004). Although acute treatment with rapamycin selectively inhibits mTORC1, prolonged treatment also causes mTORC2 inhibition (Sarbassov et al., 2006). The mTORC1 complex is composed of mTOR, Raptor, DEPTOR, PRAS40, and GβL/mLST8 (Kim et al., 2002, 2003; Wang et al., 2007; Peterson et al., 2009). The mTORC2 complex shares mTOR, DEPTOR, and GβL/mLST8 but uniquely has Rictor, mSIN1, and Protor1/2 (Dos et al., 2004; Frias et al., 2006; Pearce et al., 2007). mTORC1 phosphorylates S6 kinase and 4EBP to regulate mRNA translation (Gingras et al., 1999; Burnett et al., 1998; Choo et al., 2008; Thoreen et al., 2012; Hara et al., 1998). mTORC1 also phosphorylates and inhibits Ulk1 (ATG1 in yeast) to prevent autophagy (Ganley et al., 2009; Kim et al., 2011; Kamada et al., 2010). However, mammalian cells are largely resistant to rapamycin-dependent autophagy (Klionsky et al., 2021). Studies using rapamycin have shown that its ability to inhibit mTORC1 is substrate selective (Kang et al., 2013). In some cases, as with 4EBP, rapamycin only partially inhibits mTORC1-dependent phosphorylation and barely affects Ulk1 (Kang et al., 2013). This is one reason why rapamycin analogs (rapalogs) may have failed clinical trials for stopping cancer progression. The efficacy of rapamycin is encoded by the substrate primary sequence determinants, and alterations of select amino acids may render rapamycin-insensitive targets sensitive. The evolutionary selection of these determinants yields certain substrates less or more sensitive to nutrient-dependent phospho-regulation (Kang et al., 2013).

In addition to the incomplete inhibition of mTORC1, the clinical utility of rapalogs is limited by side effects and toxicity (Krueger et al., 2013; Li et al., 2014; Liu and Sabatini, 2020; Soefje et al., 2011). mTOR inhibitors that circumvent toxicity and cause complete mTORC1 inhibition are needed. Torin1 and PP242 were developed as ATP-competitive inhibitors that robustly inhibit mTORC1 phosphorylation of all substrates tested (Thoreen et al., 2009; Feldman et al., 2009). While Torin1 has been shown to have most of the same biological effects as rapamycin on mRNA translation, it also inhibits mTORC2 (Thoreen et al., 2012). Thus, Torin1 lacks mTORC selectivity. Bisteric inhibitors linking the ATP-competitive inhibitors to rapamycin are third-generation mTOR inhibitors (Rodrik-Outmezguine et al., 2016). For example, the rapamycin moiety in Rapalink-1 targets FKBP12 to the FKBP12 rapamycin-binding domain of mTOR but simultaneously exhibits ATP-competitive inhibition of mTORCs via MLN-0128 (Rodrik-Outmezguine et al., 2016). Rapalink-1 exhibits *in vivo* efficacy in glioblastoma and tuberous sclerosis complex (TSC) mouse models wherein mTORC1 activity is elevated (Fan et al., 2017; Mukherjee et al., 2025). However, several questions remain regarding the mechanisms by which Rapalink-1 operates, including whether it regulates transcriptomic profiles.

mTOR is located in the nucleus of diverse cell types, ranging from yeast to humans (Betz and Hall, 2013; Laplante and Sabatini, 2013). Nuclear mTOR is hypothesized to regulate transcription. As would be expected, rapamycin suppresses transcription of ribosomal proteins and metabolic pathways, including glycolysis and the tricarboxylic acid cycle, in yeast (Hardwick et al., 1999; Powers and Walter, 1999; Cardenas et al., 1999; Shamji et al., 2000). It suppresses the transcription of ribosomal genes and of metabolic pathways in yeast to prevent proliferation (Hardwick et al., 1999; Zaragoza et al., 1998). Rapamycin-dependent suppression of transcription is similar in mammalian cells and overlaps with changes caused by amino acid deprivation (Peng et al., 2002). The ability of mTOR to regulate transcription appears to be related to its presence at the promoters of Pol-I- and Pol-III-regulated regions in diverse human and murine cells, where it regulates rDNA and tRNA transcription (Mayer et al., 2004; Vazquez-Martin et al., 2011). The mTORC1 component raptor is also present in nucleoli and chromosomal nucleolus organizer regions, where it regulates rRNA transcription (Vazquez-Martin et al., 2011). It is now recognized that nuclear localized mTORC1 directly regulates transcription through multiple mechanisms (Powers and Walter, 1999; Vazquez-Martin et al., 2011; Cunningham et al., 2007; Audet-Walsh et al., 2017; Dufour et al., 2022; Claypool et al., 2004; Mayer et al., 2004). The utility of these studies is the support for the cellular roles that mTOR(C1) has in normal physiological and potentially pathophysiological states and for how inhibition could be used to treat certain diseases. Here, we examine the effects of Rapalink-1 on mTOR and transcriptomes, which may be useful for understanding its therapeutic advantages and limitations.

## RESULTS

### Rapalink-1 inhibits mTORC1 in glioblastoma cells

A previous report examined the effects of Rapalink-1 on inhibiting mTORC1 activity and proliferation in glioblastoma, and we sought to reproduce and extend these findings (Fan et al., 2017). Two well-characterized glioblastoma cell lines, LN-18 and T98G, were used. The effect of 10 nM Rapalink-1 after 3 h of treatment that reduced S6 phosphorylation was confirmed in both cell lines. S6 phosphorylation was inhibited by Rapalink-1 by ∼98% in LN-18 cells (vehicle=1.000±0.069, *N*=3, versus Rapalink-1=0.020±0.003, *N*=3) and by ∼96% in T98G cells (vehicle=1.000±0.029, *N*=3, versus Rapalink-1=0.036±0.014, *N*=3, *P*<0.0001) (Fig. 1A,B). Rapalink-1 also abrogated 4EBP Thr37/46 phosphorylation in LN-18 cells (vehicle=1.000±0.026, *N*=3, versus Rapalink-1=0.110±0.022, *N*=3) and T98G cells (vehicle=1.000±0.037, *N*=3, versus Rapalink-1=0.049±0.008, *N*=3, *P*<0.0001) (Fig. 1C,D). Rapalink-1 robustly inhibited pUlk1 (Fig. 1E,F). Rapalink-1 reduced pUlk1 by ∼90% in LN-18 cells (vehicle=1.000±0.071, *N*=3, versus Rapalink-1=0.089±0.027, *N*=3) and ∼94% in T98G cells (vehicle=1.000±0.173, *N*=3, versus Rapalink-1=0.217±0.057, *N*=3, *P*<0.01). Rapalink-1 also partially inhibited mTORC2 as indicated by an ∼80% reduction of the phospho-Ak strain transforming protein kinase (pAKT) in LN-18 (*P*<0.0001) and T98G (*P*<0.05) cells (Fig. 1G,H).

**Fig. 1.**
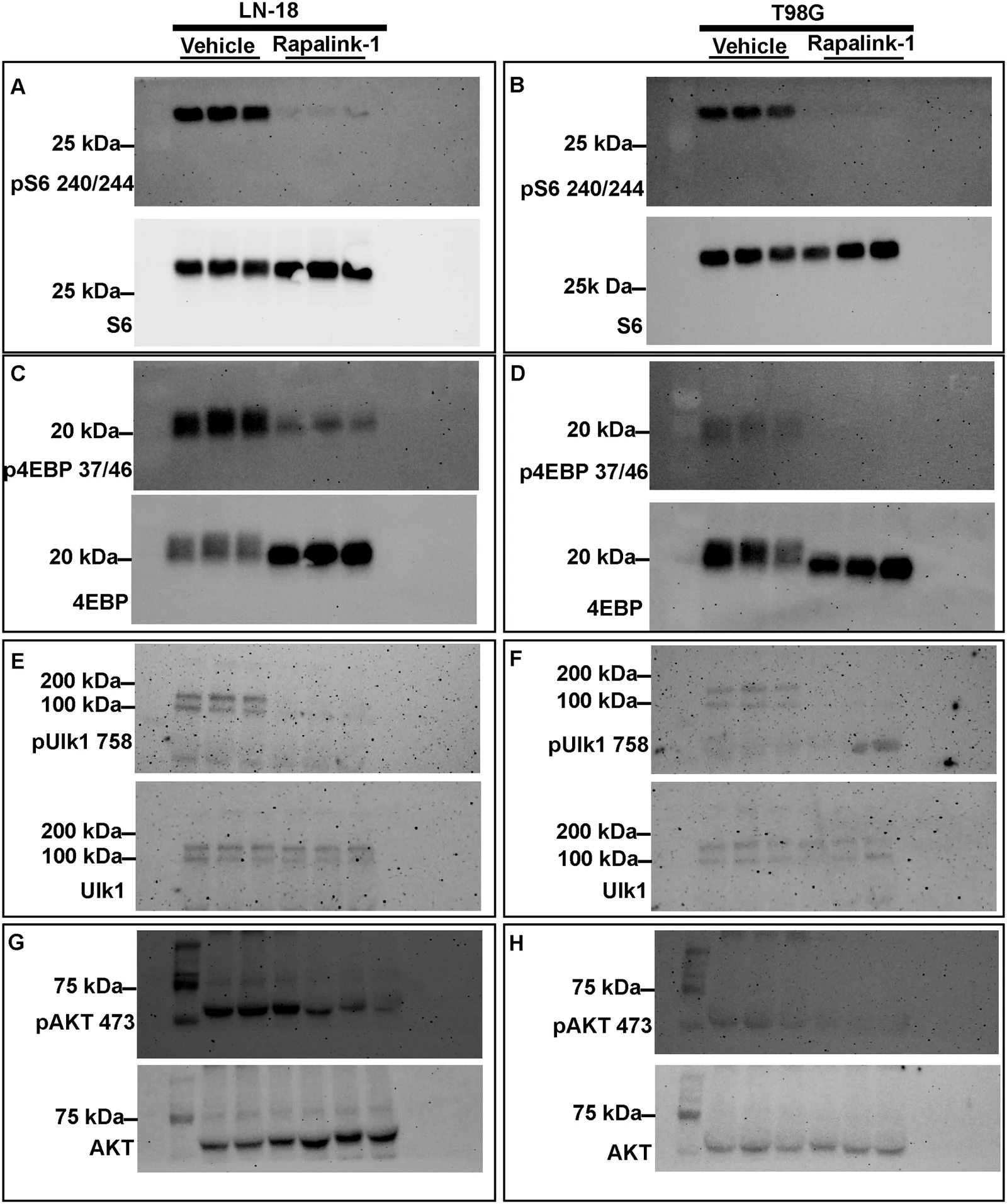
**Rapalink-1 inhibits mTORC1 in glioblastoma cells.** (A,B) LN-18 (A) or T98G (B) cells were treated with Rapalink-1 and probed for pS6 (upper) and re-probed for total S6 (lower). (C,D) LN-18 (C) or T98G (D) cells were treated with Rapalink-1 and probed for p4EBP (upper) and re-probed for total 4EBP (lower). (E,F) LN-18 (E) or T98G (F) cells were treated with Rapalink-1 and probed for pUlk1 (upper) and re-probed for total Ulk1 (lower). (G,H) LN-18 (G) or T98G (H) cells were treated with Rapalink-1 and probed for pAKT (upper) and re-probed for total AKT (lower).

### 24-h Rapalink-1 treatment inhibits neuroblastoma mTORC1 activity

Neuro-2a cells are another widely used cell line for studying mTOR. Neuro-2a mouse neuroblastoma cells are preferred due to their high transfection efficiency and intact mTORC1 pathway. The effect of long-term (24 h) Rapalink-1 treatment on mTORC1 substrate phosphorylation was subsequently examined in Neuro-2a cells. Rapalink-1 reduced the ratio of pS6/S6 by >95% (vehicle=1.000±0.1565, *N*=3, versus Rapalink-1 0.04104±0.005822, *N*=3, *P*<0.01) (Fig. 2A,B). mTORC1 phosphorylation of threonine 37/47 of 4EBP is rapamycin insensitive. The effect of Rapalink-1 on mTORC1 phosphorylation of these sites on 4EBP was therefore examined. p4EBP/4EBP was nearly completely ablated (vehicle=1.000±0.1633, *N*=3, versus Rapalink-1=0.008766±0.001791, *N*=3, *P*=0.01) (Fig. 2C,D). Total 4EBP had a notable shift in the electrophoretic mobility consistent with the inhibition of phosphorylation. We complemented our findings of glioblastoma Ulk1 and measured the ratio of pUlk1/Ulk1 in Neuro-2a cells following 24 h of Rapalink-1 treatment. Rapalink-1 reduced the ratio of pUlk1 by ∼50% (vehicle=1.000±0.1397, *N*=3, versus Rapalink-1=0.4909±0.08773, *N*=3, *P*<0.05) (Fig. 2E,F). We sought independent verification of the efficacy of Rapalink-1. We confirmed Rapalink-1 effects on pS6 (Fig. 2G-L) and p4EBP (Fig. 2M-R) using immunocytochemistry. Rapalink-1-treated cells had ∼80% less pS6 compared to vehicle-treated controls (vehicle=35.84±1.855, *n*=250, versus Rapalink-1=7.702±0.2008, *n*=250, *P*<0.0001) (Fig. 2S). p4EBP levels were reduced by Rapalink-1 treatment (vehicle=29.56±1.164, *n*=250, versus Rapalink-1=10.86±1.180, *n*=250, *P*<0.0001) (Fig. 2T). Thus, after 24 h of treatment, Rapalink-1 is an effective mTORC1 inhibitor.

**Fig. 2.**
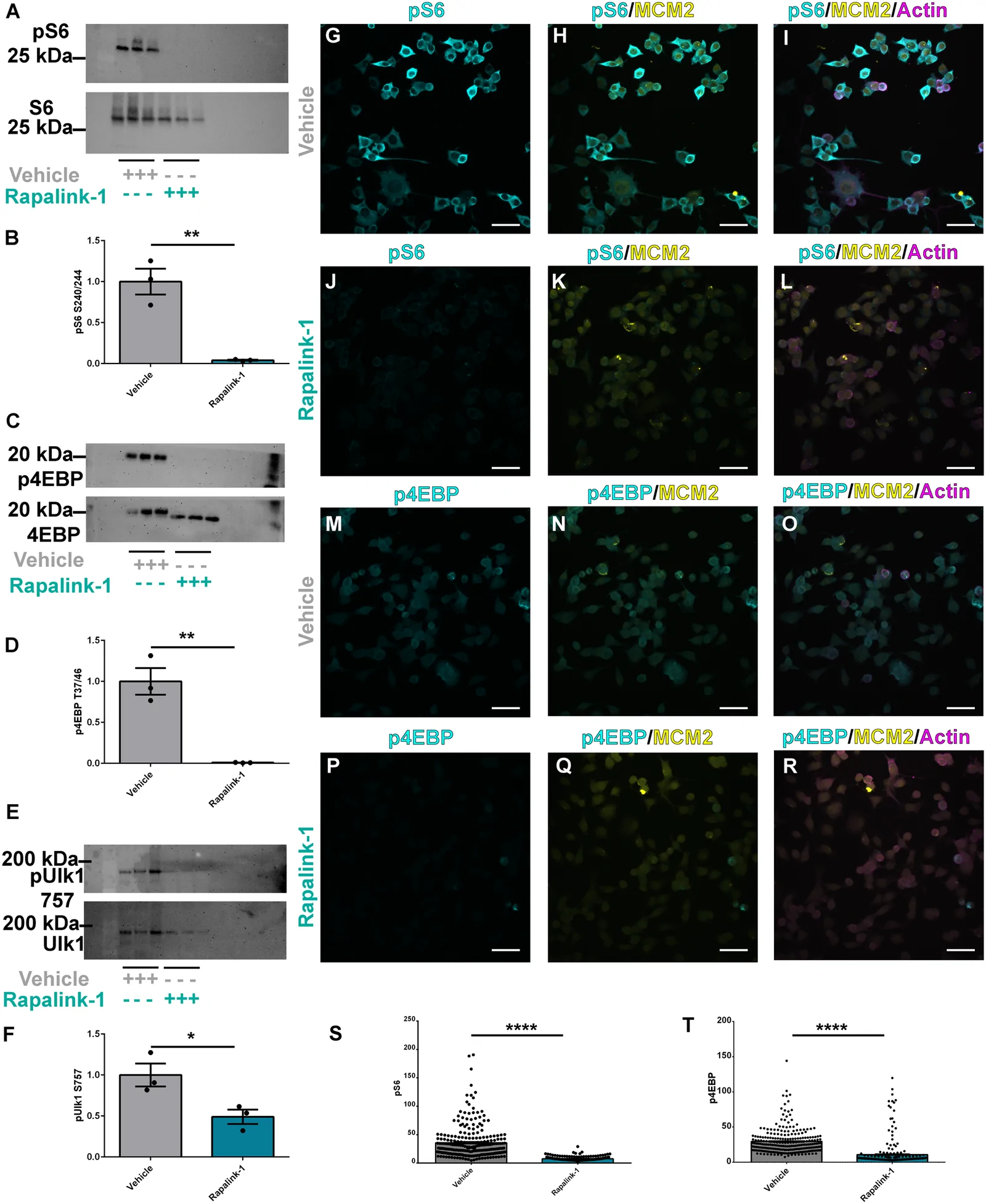
**Rapalink-1 treatment for 24 h inhibits neuroblastoma mTORC1 activity.** (A-F) Neuro-2a cells treated with 10 nM Rapalink-1 for 24 h were probed (A) and quantified (B) for pS6 and S6, probed (C) and quantified (D) for p4EBP and 4EBP, and probed (E) and quantified (F) for pUlk1 and Ulk1. All blots were probed for the phosphorylated form, stripped, and re-probed for total levels of the same proteins. (G-I) Images of Neuro-2a cells treated for 24 h with vehicle and subjected to immunocytochemistry for (G) pS6 (cyan), (H) MCM2 (yellow), and (I) actin (magenta). (J-L) Images of Neuro-2a cells treated for 24 h with 10 nM Rapalink-1 and subjected to immunocytochemistry for (J) pS6 (cyan), (K) MCM2 (yellow), and (L) actin (magenta). (M-O) Images of Neuro-2a cells treated for 24 h with vehicle and subjected to immunocytochemistry for (M) p4EBP (cyan), (N) MCM2 (yellow), and (O) actin (magenta). (P-R) Images of Neuro-2a cells treated for 24 h with 10 nM Rapalink-1 and subjected to immunocytochemistry for (P) pS6 (cyan), (Q) MCM2 (yellow), and (R) actin (magenta). (S) Quantification of pS6 in G-L. (T) Quantification of p4EBP in (M-R). *P<0.05, **P<0.01, ****P<0.0001. Scale bar: 75 μm.

### 3-h Rapalink-1 treatment inhibits neuroblastoma mTORC1 activity

Long-term (24-h) rapamycin treatment decreases the synthesis, assembly, and activity of mTORC2 (Sarbassov et al., 2006). The effect is cell line, FKB12, and mTOR dependent (Schreiber et al., 2015). Since Rapalink-1 contains a rapamycin moiety, we hypothesized that it may also destabilize mTORC2 after long-term treatment. We confirmed that Rapalink-1 inhibits the mTORC2 phosphorylation of pAKT after 24 h of treatment (vehicle=1.000±0.01046, *N*=3, versus Rapalink-1=0.3691±0.1524, *N*=3, *P*<0.05) (Fig. 3A,B). An alternative explanation of this effect could be that the MLN-0128 portion of Rapalink-1 directly binds to the mTORC2 catalytic domain. We subsequently sought to confirm the results of Rapalink-1 on Neuro-2a cells, after short-term (3 h) treatment: a timepoint that is more likely to be dependent on the MLN-0128 moiety of Rapalink-1. At this timepoint, Rapalink-1 had little to no effect on pAKT, indicating that mTORC2 assembly was not impeded (Fig. 3C,D). This result indicates that the inhibition of mTORC2 by Rapalink-1 after 24 h of treatment is likely caused by the rapamycin portion of the molecule in Neuro-2a cells; however, this is also dependent on FKBP12 levels, which are dynamically expressed (Schreiber et al., 2015). Western blot analysis of Neuro-2a lysate was performed to confirm that Rapalink-1 was an effective inhibitor after 3 h of treatment, and this was compared to the same dose and time course of treatment using rapamycin. S6 phosphorylation was inhibited by rapamycin and Rapalink-1 by ∼95% and 96%, respectively (vehicle=1.000±0.04682, *N*=3, versus Rapalink-1=0.04289±0.01891, *N*=3, versus rapamycin=0.03944±0.008318, *N*=3, *P*<0.0001) (Fig. 3E,F). There was no statistically significant difference between the efficacy of the two drugs on pS6/S6 at this dose. As previously stated, p4EBP is reported to be only partially inhibited by rapamycin. We therefore sought to confirm these findings and compare the effect of Rapalink-1 and rapamycin on p4EBP. In contrast to the effects on pS6, Rapalink-1 was superior to rapamycin and completely ablated 4EBP phosphorylation (Fig. 3G,H). Rapamycin had virtually no effect (vehicle=1.000±0.06977 versus Rapalink-1=0.01813±0.007621 versus rapamycin=1.093±0.2577, *P*<0.01) (Fig. 3G,H). Rapalink-1 was significantly different from the vehicle (*P*<0.01) and in comparison to rapamycin (*P*<0.01) (*P*-values adjusted for multiple comparisons). In comparison, rapamycin and the vehicle had similar levels of p4EBP. We further examined pUlk1 after Rapalink-1 treatment. In several experiments, Ulk1 levels often mirrored pUlk1 and were reduced by Rapalink-1. This could be a technical issue with stripping or caused by biological processes such as autophagy. We hypothesized that Rapalink-1 might induce autophagy, which suppresses Ulk1 translation and causes Ulk1 degradation (Zhong et al., 2026). To overcome problems with Rapalink-1-induced autophagy and degradation, cells were overloaded with total Ulk1 and treated with Rapalink-1 for 3 h (Fig. 3I). To address technical issues, we probed one blot for pUlk1 and stripped it and re-probed for total Ulk1, and another blot was probed only for total Ulk1. Rapalink-1 reduced pUlk1 in GFP-transfected conditions (*P*<0.001) by ∼77% and in Ulk1-transfected conditions (*P*<0.01) by 63% (*t*-test) (Fig. 3I,J). In the comparison of all conditions with GFP-transfected vehicle-treated cells, Ulk1 overexpression increased Ulk1 phosphorylation by ∼349% (*P*<0.01), and this was reversed by Rapalink-1 to levels of GFP control conditions [analysis of variance (ANOVA)] (Fig. 3J). Taken together, short-term Rapalink-1 treatment reduced mTORC1 and effectively inhibited Ulk1 phosphorylation.

**Fig. 3.**
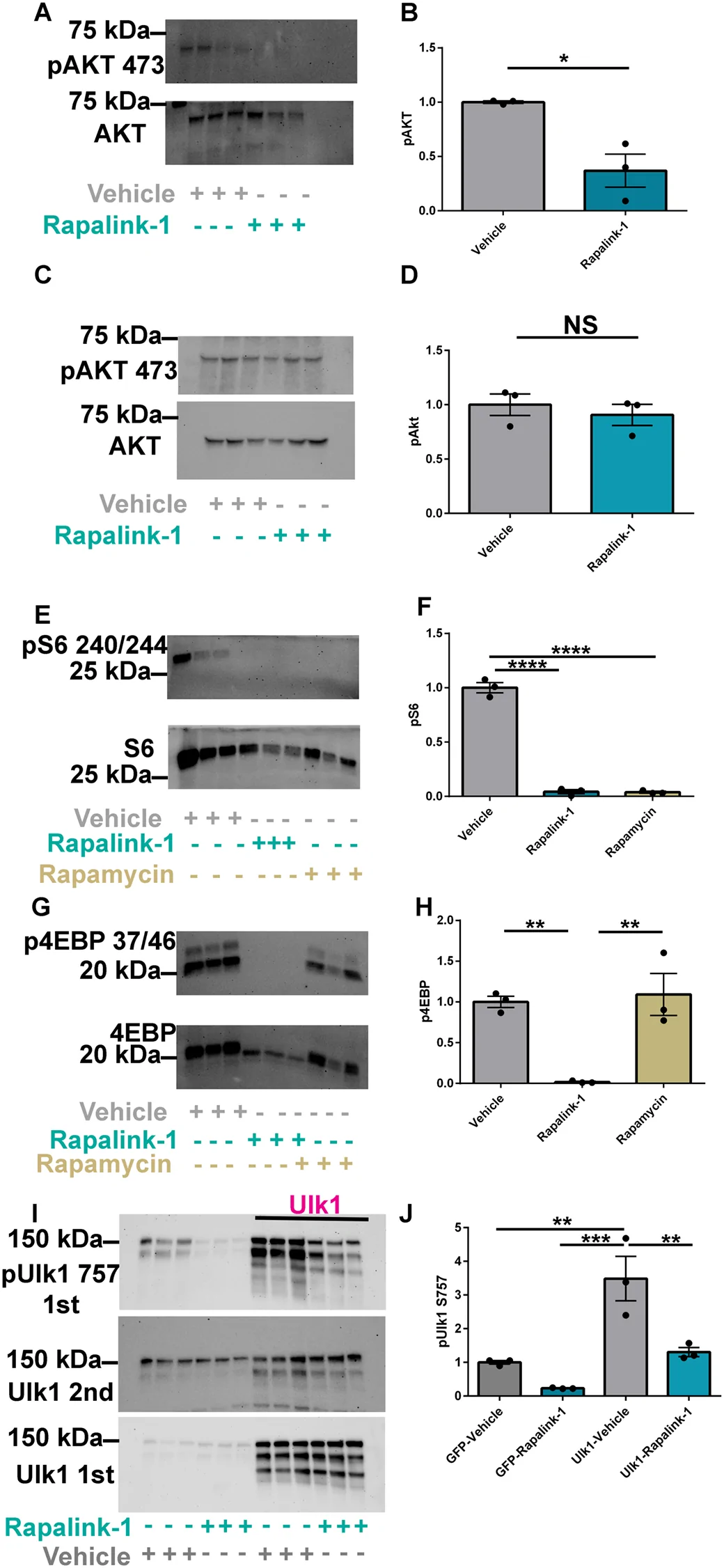
**Rapalink-1 treatment for 3 h inhibits mTORC1, not mTORC2.** (A) Neuro-2a cells treated with Rapalink-1 for 24 h and probed for pAKT (upper) and re-probed for total AKT (lower) demonstrating inhibition of mTORC2. (B) Quantification of A. (C) Neuro-2a cells treated with Rapalink-1 for 3 h and probed for pAKT (upper) and re-probed for total AKT (lower) demonstrating no effect on mTORC2. (D) Quantification of C. (E) Neuro-2a cells treated with Rapalink-1 or rapamycin for 3 h and probed for pS6 (upper) and re-probed for total S6 (lower). (F) Quantification of E. (G) Neuro-2a cells treated with Rapalink-1 or rapamycin for 3 h and probed for p4EBP (upper) and re-probed for total 4EBP (lower). (H) Quantification of G. (I) Neuro-2a cells were transfected with control plasmid or wild-type Ulk1 and, 48 h later, treated with Rapalink-1 for 3 h. Lysates were probed for pUlk1 (upper), re-probed for total Ulk1 (middle), or probed first for total Ulk1 (lower). (J) Quantification of I. *P<0.05, **P<0.01, ***P<0.001, ****P<0.0001.

We further tested the antibody specificity in Ulk1-transfected Neuro-2a cells and probed for phospho-Ulk1 and total Ulk1 with or without lambda phosphatase treatment (Fig. S1). Indeed, Ulk1 vastly increased the total and phospho-Ulk1 levels. Lambda phosphatase treatment specifically reduced only phospho-Ulk1. We also tested phospho-Ulk1 555 antibodies and confirmed this specificity. Thus, phospho-Ulk1 and total Ulk1 antibodies were reliable, confirming the specificity of effects of mTOR inhibition on pUlk1 757 and total Ulk1.

### Rapalink-1 regulation of an mTOR and p53 transcriptomic signature

While most studies have focused on the role of mTORC1 in translation, there is a growing interest in the fact that mTORC1 is present within the nucleus of diverse cells where it has a critical role in regulating transcription (Laplante and Sabatini, 2013; Vazquez-Martin et al., 2011; Audet-Walsh et al., 2017; Kim and Chen, 2000; Li et al., 2006; Zhang et al., 2002; Holmberg et al., 2025). In addition, there are distinct and reproducible transcriptomic alterations in patients with TSC that are characterized by subependymal giant cell astrocytomas and hyperactive mTORC1 signaling (Holmberg et al., 2026; Riley et al., 2023; Allavena et al., 2016). Incidentally, Rapalink-1 has modest efficacy in a TSC model (Mukherjee et al., 2025). We hypothesized that Rapalink-1 inhibition of mTOR may affect mRNA transcripts that provide valuable mechanistic insight into its potential clinical utility. Cells were therefore treated with Rapalink-1 for 3 h, RNA was isolated, and transcriptomes were sequenced. Consistent with our expectations, Neuro-2a cells treated with Rapalink-1 underwent robust transcriptomic changes as illustrated in the heatmap (Fig. 4A). There were 1307 transcripts differentially expressed with a *P*adj<0.01. Of those, 488 were reduced by a log_2_ fold change of >−0.5, and 468 were increased by a log_2_ fold change of ≥0.5. We subjected all downregulated transcripts (Fig. 4B) and the top downregulated transcripts (log_2_≥1.0, Fig. 4C) to bioinformatic analysis using multiple programs, including String pathway analysis, Gene Ontology (GO) analysis, and GProfiler analysis using Metascape (Tables S1-S3). As expected, significantly downregulated transcripts were representative of changes to the TOR pathway (Fig. 4C; Tables S1 and S2). This was exemplified by reduced levels of *ei4EBP2* mRNA. Other examples include reduced transcript levels of *Sestrin2*, *Castor*, *Rps6ka1*, *Ddit1*, and *Larp1*, some of which have predicted p53 binding sites. Rapalink-1 reduced transcripts of genes associated with GO terms consistent with the role of mTORC1 in brain development, including negative regulation of neuron projection morphogenesis (*P*<Log10−9.72), brain development (*P*<Log10−6.69), and nerve development among others (*P*<Log10−5.71). While mTOR pathway signaling would be expected to be altered, the fact that there were changes at the mRNA level indicates that there may be feedback loops that allow for changes in mTOR kinase activity to be transformed into alterations in transcriptomes. These transcriptomic changes positively confirmed the cell type used, Neuro-2a cells, as determined by the Pattern Gene Database (PaGenBase), as identified through Metascape (Fig. 4D). p53 consensus sequences were identified in many downregulated transcripts using the Transcriptional Regulatory Relationships Unraveled by Sentence-based Text Mining (TRRUST) (Fig. 4E). Treatment with Rapalink-1 also upregulated mRNA transcripts. Many upregulated pathways appeared to be related to immunologically relevant and apoptotic signaling pathways: tumor necrosis factor signaling pathway (mmu04668) (*P*<Log10−6.71), response to interleukin-1 (GO:0070555) (*P*<Log10−5.83), cytokine-mediated signaling pathway (GO:001959) (*P*<Log10−2.64), regulation of extrinsic apoptotic signaling pathway via the death domain receptor (GO:1902041) (*P*<Log10−2.62), and positive regulation of the apoptotic process (GO:0043065) (*P*<Log10−5.72). These results are consistent with the pro-survival role of mTOR in the nervous system. A seemingly convergent mechanism relating these pathways is regulation by p53. Given that many processes are regulated by p53 and that downregulated transcripts had p53 consensus sequences in the promoter, Rapalink-1 may also change p53 levels. To test this hypothesis, Neuro-2a cells were treated with Rapalink-1 for 3 h, and a western blot analysis was performed. p53 protein expression was reduced (*P*<0.01) by Rapalink-1 (Fig. 4F). Taken together, these results demonstrate that Rapalink-1 causes an mTOR- and p53-regulated transcriptome.

**Fig. 4.**
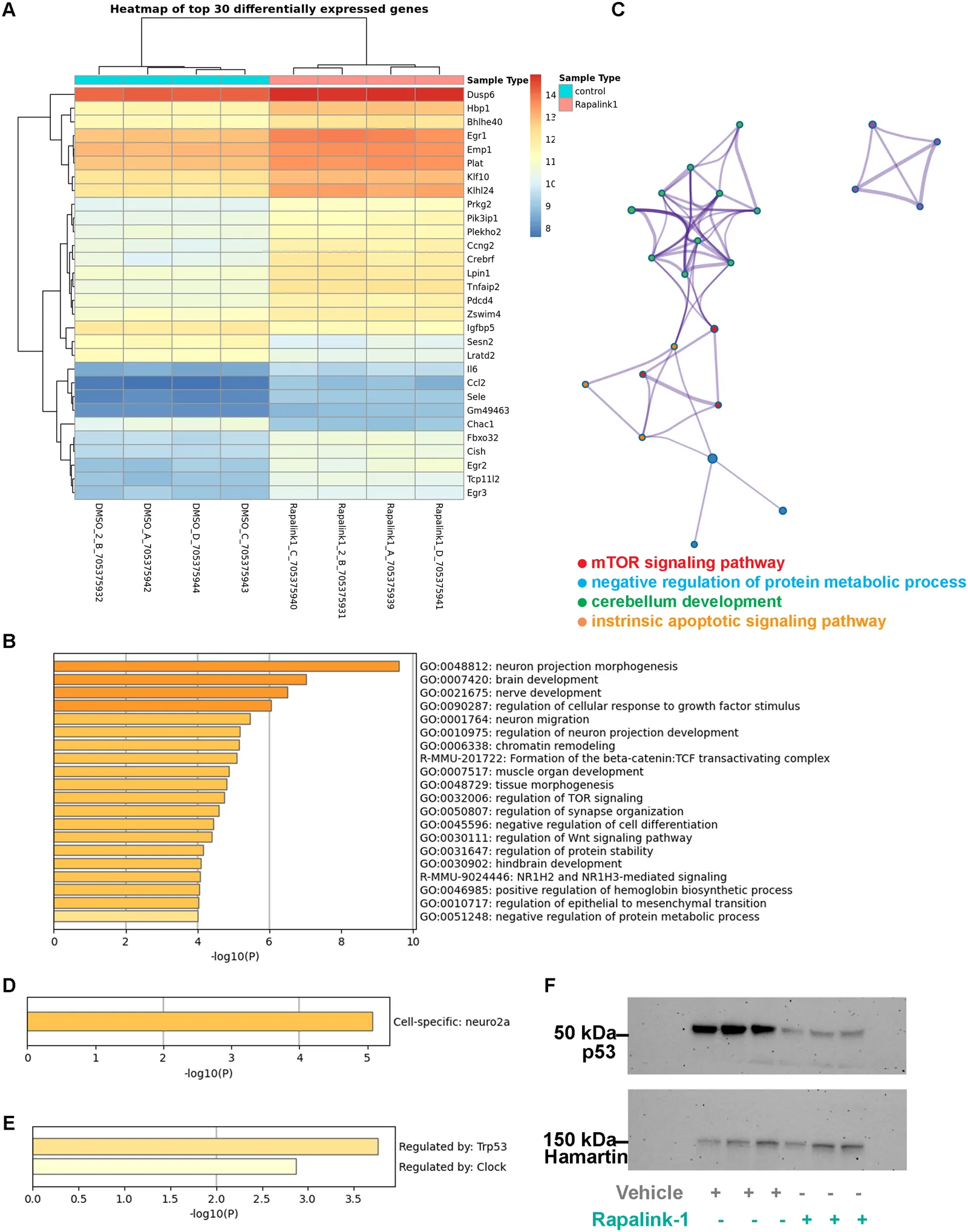
**Rapalink-1 regulation of an mTOR and p53 transcriptomic signature.** Neuro-2a cells were treated with vehicle or 10 nM Rapalink-1 for 3 h and subjected to RNA sequencing analysis to identify differentially expressed transcripts and enriched pathways. (A) Heatmap of the top 30 differentially expressed genes. (B) Significantly enriched GO terms among downregulated transcripts searched using Metascape. (C) Topmost decreased transcript enrichment network from Metascape demonstrating representation of mTOR signaling. Each node (circle) represents an enriched GO term. Node color indicates the functional cluster, node size is proportional to the number of input transcripts associated with the term, and edges (lines) connect GO terms that share a significant number of input transcripts. (D) Summary of enrichment analysis in the PaGenBase, as identified through Metascape. (E) Summary of enrichment analysis from Metascape in TRRUST. (F) Neuro-2a cells treated with Rapalink-1 for 3 h and probed for p53 (upper) and re-probed for hamartin/TSC1 (lower).

## DISCUSSION

Here, Rapalink-1 was confirmed as an effective inhibitor of mTOR, and several novel mechanisms of action were identified. The findings were first examined in reference to two glioblastoma cell lines: LN-18 and T98G cells. Both cell lines exhibited robust responses to Rapalink-1, including a strong inhibition of mTORC1-mediated S6, 4EBP, and Ulk1 phosphorylation. This is particularly relevant because Ulk1 is a master regulator of autophagy and mTORC1 phosphorylation of Ulk1 prevents autophagy. Rapamycin does not inhibit mTORC1 phosphorylation of Ulk1 or robustly induce autophagy and only partially inhibits 4EBP phosphorylation. Based on these results, we agree with the conclusion that Rapalink-1 may offer advantages over rapamycin with respect to treating glioblastoma.

Neuro-2a cells are used to model neuroblastoma and study mTOR signaling. Therefore, the effects of prolonged exposure of Neuro-2a cells to Rapalink-1 were subsequently studied. Rapalink-1 treatment for 24 h led to nearly complete inhibition of S6 and 4EBP. Given the strength of these results, the efficacy of Rapalink-1 was confirmed by immunocytochemistry. The findings on the effect of Rapalink-1 on Ulk1 were also extended to Neuro-2a cells and confirmed that Rapalink-1 reduced mTORC1 phosphorylation of Ulk1.

Rapamycin acts by binding to FKBP12, altering its conformation and promoting its association with mTORC1, thereby inhibiting its activity. The dissociation of the rapamycin–FKBP12 complex is relatively slow and occurs in the absence of additional mTORC components. Consequently, prolonged rapamycin treatment can impair the efficient assembly of mTORC2 due to its persistent binding to mTOR, resulting in reduced mTORC2-mediated phosphorylation of AKT (Sarbassov et al., 2006). Because Rapalink-1 contains a rapamycin moiety, we hypothesized that a similar effect might occur. Indeed, we observed that AKT phosphorylation was reduced following 24 h of Rapalink-1 treatment. To address potential effects on mTORC2 assembly, we performed additional experiments for 3 h and compared the effects of Rapalink-1. Acute Rapalink-1 treatment had little effect on AKT phosphorylation in Neuro-2a cells. These findings suggest that the effect of 24-h Rapalink-1 treatment on AKT may be due to the partial inhibition of the mTORC2 assembly, whereas at this dose and after 3 h of treatment, the MLN-0128 has less of an effect on mTORC2. However, this is also controlled by the level of FK-506-binding proteins (Schreiber et al., 2015). In contrast to rapamycin, which inhibited only mTORC1-mediated phosphorylation of S6, Rapalink-1 effectively inhibited phosphorylation of multiple mTORC1 substrates, including 4EBP, S6, and Ulk1.

Occasionally, phospho-Ulk1 levels mirrored total Ulk1 levels, suggesting that mTORC1-mediated phosphorylation of Ulk1 may regulate its stability. Previous studies have shown that Ulk1, a key regulator of autophagy, is degraded during nutrient deprivation (Zhong et al., 2026). It was therefore hypothesized that effective inhibition of Ulk1 might promote autophagy and subsequent Ulk1 degradation. These findings led us to hypothesize that Ulk1 degradation by autophagy machinery could be mitigated by overexpressing Ulk1 prior to Rapalink-1 treatment. This approach allowed us to distinguish the effects of Rapalink-1 on total Ulk1 levels and directly demonstrate its inhibition of Ulk1 phosphorylation. Antibody specificity was confirmed by Ulk1 overexpression and lambda phosphatase treatment. Both total and phospho-Ulk1 antibodies were specific.

Given that mTOR complexes regulate numerous substrates, including nuclear targets involved in transcriptional control, we investigated the extent to which Rapalink-1 alters the transcriptome of Neuro-2a cells. We observed robust transcriptomic changes within 3 h of treatment. As expected, multiple analyses indicated that Rapalink-1 reduced the abundance of transcripts associated with the mTOR pathway. Additionally, pathways involved in neuronal development were significantly downregulated, including the Wnt signaling pathway, as well as GO terms such as neuron projection morphogenesis, neuron migration, and nerve development.

In contrast, the upregulated transcripts were associated with diverse cellular pathways, most notably the positive regulation of programmed cell death. We also observed enrichment of pathways related to interleukin-1 response, cytokine-mediated signaling, RAF-independent MAPK1/3 activation, and other immune-related processes. Notably, many of these transcripts contain p53 binding sites and are associated with an increase in the senescence-associated secretory phenotype. We therefore examined whether markers of senescence were also changed. We identified a reduction in p53 caused by Rapalink-1. Although the underlying mechanism remains unclear, mTOR-dependent regulation of senescence appears to play an important role in mTORC1-related genetic disorders (Ribierre et al., 2024). Torin1 also increases p53 phosphorylation and quiescence in induced pluripotent stem cells that have been differentiated into cardiomyocytes (Garbern et al., 2020). However, given the difference in directionality, these results indicate that the change in p53 is most likely indirectly affected by mTORC1 inhibition. The transcriptional changes identified in this study may be shared or distinct from those caused by rapamycin. Future studies should define whether any and if so which transcriptomic effects of Rapalink-1 are dependent on the rapamycin or MLN-0128 moieties, and whether these effects are completely distinct.

Taken together, the results presented here corroborate and expand upon the effects of Rapalink-1 on mTOR, including its ability to inhibit Ulk1, and to modulate transcriptomes, which may have implications for its future clinical application.

## MATERIALS AND METHODS

### Experimental model and subject details

#### Cell culture and transfection

Neuro-2a mouse neuroblastoma cells (American Type Culture Collection CCL-131), LN-18 cells (American Type Culture Collection CRL-2610), and T98G cells (American Type Culture Collection CRL-1690) were grown in polystyrene tissue-culture-treated multi-well plates or flasks (Falcon; BD Biosciences Discovery Labware) in a 5% CO_2_ incubator at 37°C. The cells were routinely propagated in DMEM with 10% FBS and 1× penicillin/streptomycin. DNA transfections were performed with PolyJet (SignaGen Laboratories) 24 h after seeding. DNA plasmids were CMV-Ulk1 from Addgene (plasmid #27629, pcdna6.2-myc Ulk1) and CAG-GFP from Addgene (plamid #11150). PolyJet was mixed with DMEM and DNA and then added to the cultures.

#### Pharmacological treatments

The cells were treated with the indicated amounts of rapamycin (#9904s, Cell Signaling Technology) or Torin1 (#14379, Cell Signaling Technology) or Rapalink-1 (ApexBio #A8764). The compounds were diluted in DMSO (MP Biomedicals). Equimolar DMSO was used for all conditions. The cells were treated with DMSO, rapamycin, Torin1, or Rapalink-1 at the dose and times indicated in the figures.

#### Western blotting

Samples were collected in 2% weight/volume (w/v) SDS in radioimmunoprecipitation assay (RIPA) buffer containing protease and phosphatase inhibitor cocktail. The lysates were briefly sonicated on ice and centrifuged at 17,115 ***g*** for 5 min in a tabletop Eppendorf 5414 centrifuge. Samples were briefly sonicated at maximum amplitude (100%, Qsonica) for 10 s, three times, with 30-s resting intervals. The lysate was then transferred to a fresh reaction tube on ice and centrifuged at 21,130 ***g*** for 15 m in a tabletop Eppendorf 5415 centrifuge at 4°C. The protein concentration was quantified using the Pierce Micro BCA assay. Equal amounts of protein were brought to equal volumes with lysis buffer, as described above, and Laemmli buffer before being heated to 95°C for 5 min. The proteins were resolved using standard electrophoresis conditions on 10-12% polyacrylamide precast mini-PROTEAN TGX gels and transferred to polyvinylidene difluoride (PVDF) membranes. The membranes were rinsed in TBST (Tris-buffered saline with 0.1% Tween 20) for 5 min at room temperature and subsequently blocked in either 5% w/v nonfat milk or 2% w/v BSA (for the phospho-AKT blot only) in TBST for 1 h at room temperature. Samples were incubated overnight at 4°C with the following antibodies: rabbit anti-phospho-S6 ribosomal protein (1:2500; Cell Signaling Technology; Ser240/244; 61H9; #4838), rabbit anti-total S6 ribosomal protein (1:5000; Cell Signaling Technology; 5G10; #2217), rabbit anti-phospho-4EBP1 (1:1000; Cell Signaling Technology; Thr37/46; 236B4; #2855), rabbit anti-total 4EBP1 (1:5000; Cell Signaling Technology; 53H11; #9644), rabbit anti-phospho-Ulk1 757 (1:1000; Cell Signaling Technology; Ser757; D7O6U, #14202), rabbit anti-phospho-Ulk1 555 (1:1000; Cell Signaling Technology; Ser555; D1H4; #5869), rabbit anti-total Ulk1 (1:1000; Cell Signaling Technology; D8H5, #8054), rabbit anti-phospho-AKT 473 (1:1000; Cell Signaling Technology; 736E11; Ser473; #4060), rabbit anti-total AKT (pan) (1:1000; Cell Signaling Technology; C67E7, #4691), mouse anti-p53 (1:1000; Cell Signaling Technology; 1C12, #2524), rabbit anti-LC3B (1:1000; Cell Signaling Technology; D11, #3868), and rabbit anti-TSC1 (1:1000; Cell Signaling Technology; D43E2; #6935). Following three additional 5-min rinses in TBST, the samples were incubated for 1 h at room temperature with either a donkey anti-rabbit (DKXRBHRP HIGH XADS; 1:5000; Invitrogen; SA1-200) or a donkey anti-mouse (DKXMOHRP HIGH XADS; 1:1000; NOVEX; A16017) secondary antibody in blocking buffer, then subjected to three 15-min washes in TBST, and visualized using enhanced chemiluminescence with film. All phospho-antibody/total antibody ratios were quantified using the same PVDF membrane. The PVDF membranes were briefly stripped for 20-25 m at room temperature using Restore Western Blot Stripping Buffer (#21059, Thermo Fisher Scientific) before re-probing for total levels of that protein. Western blots were imaged using a Bio-Rad ChemiDoc MP (Bio-Rad, Hercules, CA).

#### Phosphatase experiments

The Neuro-2a cells were transfected, and the lysate was collected for western blot as described above. Following the transfer, the PVDF membrane was cut in half, so both halves contained six lanes each (three control and three Ulk1). One of these halves was subjected to lambda protein phosphatase (Sigma-Aldrich; P9614) treatment for 4 h, followed by an alkaline phosphatase (Sigma-Aldrich; P0114) treatment for an additional 30 min. The blots were then washed, blocked and probed as described above.

#### Immunocytochemistry

Cells cultured on a Fisherbrand microscope cover glass (Fisher Scientific) were removed from the incubator and immediately incubated with an equal volume of 4% paraformaldehyde (PFA) in complete media for a final concentration of 2% PFA. The medium was then removed and exchanged for 4% PFA in complete media and incubated at 37°C in the CO_2_ incubator for 30 min. The cells were then rinsed with 0.1% Tween 20 in DMEM for 15 min at room temperature. The cells were then rinsed three times in 0.1% Tween 20 in PBS at room temperature for 15 min each wash. The cells were then incubated in a solution of 2% BSA weight/volume, 0.1% Triton X-100, and 0.1% Tween 20 in PBS for 1 h at room temperature. The cells were then incubated overnight at 4°C in primary antibodies (anti-phospho-S6 ribosomal protein: 1:1000; Cell Signaling Technology; Ser 240/244; 61H9; #4838, anti-phospho-4E-BP1: 1:1000; Cell Signaling Technology; Thr37/46; Cat# 236B4), with MCM2 (N-19) (1:1000; Santa-Cruz Biotechnology; Cat# sc-9839) and beta-Actin (13E5) (1:1000; Cell Signaling Technology Cat# 4970), with 2% BSA and 0.1% Tween 20 in PBS. The cells were subjected to three additional washes in PBS containing 0.1% Tween 20. The cells were then incubated with the appropriate secondary antibody (Alexa Fluor series; 1:500; Invitrogen) in 2% BSA and 0.1% Tween 20 in PBS overnight at 4°C. The sections were mounted in ProLong Antifade Mountant (Thermo Fisher Scientific). Images were acquired using a spectral confocal microscope (Leica SPE) with a ×20 dry objective (NA 0.75).

#### Image analysis

Images of cells (×20) were uploaded to Fiji (ImageJ 1.5 g) and subjected to region-of-interest measurements. The freehand selection tool was used to trace the cell somas as outlined by actin. The mean gray values for phosphorylated mTOR substrates were then measured. Actin and MCM2 intensity were also measured by scrolling through the appropriate channel. The values were then inserted into Excel, and all values were normalized to the specific phospho-substrate in vehicle (DMSO)-treated conditions.

#### Transcriptomic analysis

TRIzol was added to Neuro-2a cell cultures following the removal of media containing vehicle or Rapalink-1 (*N*=4 DMSO-treated and *N*=4 Rapalink-1-treated biological replicates). The lysate was removed, triturated, and placed on ice in pre-chilled 1.5 ml reaction tubes. Chloroform was added to tubes and vigorously shaken by hand for 15 s. The samples were incubated for 5 min at room temperature and then centrifuged at 12,000 ***g*** at 4°C. The upper aqueous phase was placed in a fresh tube. The aqueous phase was used to precipitate RNA by adding isopropanol and incubated overnight at −20°C. The samples were then removed and centrifuged for 10 min at 12,000 ***g*** at 4°C. The supernatant was removed, and the pellets were washed with 75% ethanol. The samples were spun at 7500 ***g*** for 5 min at 4°C. The supernatant was removed, and the pellet was air-dried and resuspended in dH_2_O. The samples were incubated with DNase I and buffer RDD. The samples were then subjected to a Qiagen MinElute column cleanup using an RNeasy MinElute spin column. Briefly, buffer RLT was added to the samples and mixed, followed by the addition of 100% ethanol. The samples were then mixed and transferred to an RNeasy MinElute spin column in a collection tube. The samples were centrifuged for 15 s at 8000 ***g*** twice. The columns were placed into a fresh 2 ml collection tube, and 80% ethanol was added to the MinElute column. The samples were centrifuged for 2 min at 8000 ***g***, and the spin column was placed into a new tube. The samples were subjected to an additional centrifugation, and the RNA was eluted in RNase-free water and subjected to quantitative and qualitative analyses. RNA concentrations and purity were assessed using a NanoDrop Lite Spectrophotometer (Thermo Fisher Scientific). RNA concentration and quality were verified by a Qubit assay and Agilent TapeStation analysis, which confirmed the integrity of the RNA. Libraries were prepared using Universal Plus mRNA-Seq with a NuQuant (Tecan 0520-24) kit according to the manufacturer's protocol. The samples were sequenced using 150-base-pair end reads on an Illumina NovaSeq 6000 with a NovaSeq S1 flow cell. The samples were processed using the Dragen Pipeline (v4.3.13) with Illumina BaseSpace and Dragen Differential Expression. A heatmap of the top 30 genes based on adjusted *P*-values was subjected to bi-cluster analysis to check for correlation between samples. Bioinformatics was executed using the Metascape (https://metascape.org) pipeline to identify differentially enriched GO terms and transcription factor binding sites of downregulated transcripts, as indicated in the results. The search for upregulated GO terms used a base mean of >100 and log_2_>1 and inserted the 147 transcripts into Metascape with the default settings.

### Quantification and statistical analysis

The measurements were graphed, and a statistical analysis was performed using GraphPad Prism software (Version 8.2.0, GraphPad Software Inc.). Statistical significance was determined using one-way ANOVA with Tukey's multiple-comparisons test or Student's *t*-test. *N* (number of cultures) and *n* (number of cells) are listed where applicable. Error bars are reported as the standard error of the mean.

## Supplementary Material

10.1242/biolopen.062902_sup1 Supplementary information
