# Supplementary material for: Rapalink-1 reveals an mTOR- and p53-regulated transcriptional program in Neuro-2a cells
Source: Biol Open. 2026 Oct 22;15(10):bio062902. doi: 10.1242/bio.062902 (PMC13730132; doi:10.1242/bio.062902)
Supplement: Supplementary file 1 [file biolopen-15-062902-s1.pdf]

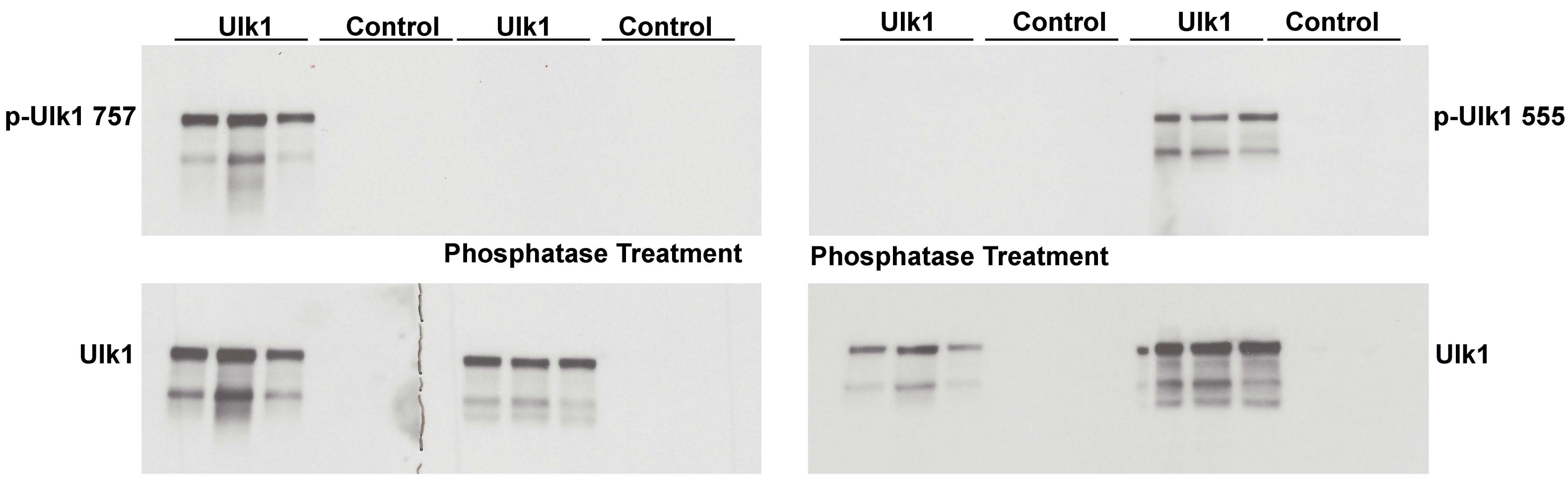

Fig. S1. Confirmation of Ulk1 antibody specificity. Neuro-2a cells were transfected with control or Ulk1 plasmids and lysates were subjected to western blot. Half of the blot was subjected to lambda phosphatase treatment and then probed for either pUlk1 757 or pUlk1 555. Blots were then stripped and re-probed for total Ulk1.

Biology Open • Supplementary information

Table S1. Results of enrichment analysis. Metascape generated search results for down-regulated transcripts, providing GO terms, Reactome Gene Sets, WikiPathways, and KEGG Pathway results.

Down-Regulated

GO	Category	Description	Count	%	Log10(P)	Log10(q)
GO:0048812	GO Biological Processes	neuron projection morphogenesis	38	8.21	-9.72	-5.65
GO:0007420	GO Biological Processes	brain development	40	8.64	-6.69	-3.29
GO:0021675	GO Biological Processes	nerve development	12	2.59	-5.71	-2.37
GO:0090287	GO Biological Processes	regulation of cellular response to growth factor stimulus	22	4.75	-5.56	-2.35
GO:0001764	GO Biological Processes	neuron migration	15	3.24	-5.51	-2.35
GO:0010975	GO Biological Processes	regulation of neuron projection development	30	6.48	-5.24	-2.16
GO:0006338	GO Biological Processes	chromatin remodeling	33	7.13	-5.23	-2.16
R-MMU-201722	Reactome Gene Sets	Formation of the beta-catenin:TCF transactivating complex	6	1.3	-5.11	-2.08
GO:0007517	GO Biological Processes	muscle organ development	19	4.1	-4.93	-1.91
GO:0048729	GO Biological Processes	tissue morphogenesis	33	7.13	-4.88	-1.89
GO:0032006	GO Biological Processes	regulation of TOR signaling	14	3.02	-4.78	-1.86
GO:0050807	GO Biological Processes	regulation of synapse organization	23	4.97	-4.65	-1.75
GO:0045596	GO Biological Processes	negative regulation of cell differentiation	35	7.56	-4.52	-1.65
GO:0030111	GO Biological Processes	regulation of Wnt signaling pathway	20	4.32	-4.44	-1.63
GO:0031647	GO Biological Processes	regulation of protein stability	21	4.54	-4.21	-1.46
R-MMU-9024446	Reactome Gene Sets	NR1H2 and NR1H3-mediated signaling	4	0.86	-4.1	-1.41
GO:0051248	GO Biological Processes	negative regulation of protein metabolic process	32	6.91	-4.06	-1.41
GO:0010717	GO Biological Processes	regulation of epithelial to mesenchymal transition	10	2.16	-4.06	-1.41
GO:0046985	GO Biological Processes	positive regulation of hemoglobin biosynthetic process	3	0.65	-4.06	-1.41
WP113	WikiPathways	TGF beta signaling pathway	7	1.51	-4.02	-1.38

Table S2. Results of enrichment analysis. Metascape generated search results for topmost down-regulated transcripts, providing GO terms, Reactome Gene Sets, WikiPathways, and KEGG Pathway results.

Top most Down-Regulated

GO	Category	Description	Count	%	Log10(P)	Log10(q)
mmu04150	KEGG Pathway	mTOR signaling pathway - Mus musculus (house mouse)	6	12.5	-5.95	-1.71
R-MMU-4641263	Reactome Gene Sets	Regulation of FZD by ubiquitination	3	6.25	-4.98	-1.04
GO:0099150	GO Biological Processes	regulation of postsynaptic specialization assembly	3	6.25	-4.48	-0.74
GO:0051248	GO Biological Processes	negative regulation of protein metabolic process	8	16.67	-3.78	-0.23
GO:0021549	GO Biological Processes	cerebellum development	4	8.33	-3.69	-0.22
GO:0001942	GO Biological Processes	hair follicle development	3	6.25	-2.89	0

GO:0007416	GO Biological Processes	synapse assembly	3	6.25	-2.6	0
GO:0008219	GO Biological Processes	cell death	6	12.5	-2.41	0

Table S3. Results of enrichment analysis. Metascape generated search results for up-regulated transcripts, providing GO terms, Reactome Gene Sets, WikiPathways, and KEGG Pathway results.

Up-Regulated

GO	Category	Description	Count	%	Log10(P)	Log10(q)
mmu04668	KEGG Pathway	TNF signaling pathway - Mus musculus (house mouse)	8	6.9	-6.71	-2.47
GO:0070555	GO Biological Processes	response to interleukin-1	7	6.03	-5.83	-1.95
GO:0043065	GO Biological Processes	positive regulation of apoptotic process	15	12.93	-5.72	-1.95
R-MMU-112409	Reactome Gene Sets	RAF-independent MAPK1/3 activation	4	3.45	-5.32	-1.79
WP5128	WikiPathways	Fibrin complement receptor 3 signaling pathway	4	3.45	-4.09	-1.12
GO:2000351	GO Biological Processes	regulation of endothelial cell apoptotic process	4	3.45	-3.62	-0.82
GO:0141124	GO Biological Processes	intracellular signaling cassette	13	11.21	-3.58	-0.82
GO:0007623	GO Biological Processes	circadian rhythm	6	5.17	-3.57	-0.82
mmu04625	KEGG Pathway	C-type lectin receptor signaling pathway - Mus musculus (house mouse)	5	4.31	-3.52	-0.78
GO:0035914	GO Biological Processes	skeletal muscle cell differentiation	4	3.45	-3.31	-0.64
GO:0007033	GO Biological Processes	vacuole organization	6	5.17	-2.92	-0.44
GO:0034103	GO Biological Processes	regulation of tissue remodeling	4	3.45	-2.92	-0.44
GO:0001959	GO Biological Processes	regulation of cytokine-mediated signaling pathway	5	4.31	-2.64	-0.29
GO:1902041	GO Biological Processes	regulation of extrinsic apoptotic signaling pathway via death domain receptors	3	2.59	-2.62	-0.28
GO:0010718	GO Biological Processes	positive regulation of epithelial to mesenchymal transition	3	2.59	-2.42	-0.15
GO:0031667	GO Biological Processes	response to nutrient levels	9	7.76	-2.34	-0.1
mmu04068	KEGG Pathway	FoxO signaling pathway - Mus musculus (house mouse)	4	3.45	-2.28	-0.07
GO:0048662	GO Biological Processes	negative regulation of smooth muscle cell proliferation	3	2.59	-2.18	-0.01
mmu04820	KEGG Pathway	Cytoskeleton in muscle cells - Mus musculus (house mouse)	5	4.31	-2.12	0
mmu05418	KEGG Pathway	Fluid shear stress and atherosclerosis - Mus musculus (house mouse)	4	3.45	-2.11	0

Biology Open • Supplementary information
